# FaceMask: A New Image Dataset for the Automated Identification of People Wearing Masks in the Wild

**DOI:** 10.3390/s22030896

**Published:** 2022-01-24

**Authors:** Michalis Vrigkas, Evangelia-Andriana Kourfalidou, Marina E. Plissiti, Christophoros Nikou

**Affiliations:** 1 Department of Communication and Digital Media, University of Western Macedonia, 52100 Kastoria, Greece; mvrigkas@uowm.gr; 2 Department of Computer Science & Engineering, University of Ioannina, 45110 Ioannina, Greece; evalinakrf@gmail.com (E.-A.K.); marina@uoi.gr (M.E.P.)

**Keywords:** face-mask, mask detector, dataset, neural networks, Darknet, YOLO

## Abstract

The rapid spread of the COVID-19 pandemic, in early 2020, has radically changed the lives of people. In our daily routine, the use of a face (surgical) mask is necessary, especially in public places, to prevent the spread of this disease. Furthermore, in crowded indoor areas, the automated recognition of people wearing a mask is a requisite for the assurance of public health. In this direction, image processing techniques, in combination with deep learning, provide effective ways to deal with this problem. However, it is a common phenomenon that well-established datasets containing images of people wearing masks are not publicly available. To overcome this obstacle and to assist the research progress in this field, we present a publicly available annotated image database containing images of people with and without a mask on their faces, in different environments and situations. Moreover, we tested the performance of deep learning detectors in images and videos on this dataset. The training and the evaluation were performed on different versions of the YOLO network using Darknet, which is a state-of-the-art real-time object detection system. Finally, different experiments and evaluations were carried out for each version of YOLO, and the results for each detector are presented.

## 1. Introduction

In recent years, the COVID-19 pandemic has unexpectedly emerged, causing the deaths of millions of people in all countries of the world. Almost two years since the first appearance of the pandemic, COVID-19 is not fully defeated and it still negatively influences our lives. Personal hygiene is the main measure to prevent the spread of the virus and the use of a mask is considered necessary and even mandatory in many countries.

While the automated identification and classification of faces in realtime, based on deep learning techniques, have concerned many researchers, the problem of identifying people wearing a mask in crowded places is not sufficiently resolved yet [1]. One of the reasons for this fact is the lack of the existence of annotated image datasets, which is a prerequisite for the training of deep learning algorithms.

For face-identification algorithms to work well, it is a prerequisite that they are trained and tested on a large set of collected images. Moreover, such images should also be captured under different lighting conditions and at different viewpoints [2]. The largest collections have been gathered online. A dataset, called MegaFace, consists of 3.3M facial images gathered from Flickr [3], while the well-known dataset MSCeleb [4] consists of 10M images of nearly 100,000 individual subjects, collected from the Web.

In this work, we present a systematic process for the construction of a new image dataset and we introduce the novel publicly available image dataset FaceMask, which consists of 4866 annotated images. To stimulate further research, we will make the data publicly available [5]. The images contain people wearing a mask and people without a mask in indoor and public places. This dataset is used for the training and evaluation of automated techniques for the detection of human faces and their classification into two categories, faces with a mask and faces without a mask. The algorithms are based on different versions of YOLO [6] using the Darknet [7]. More specifically, we trained the YOLOv3 [8], YOLOv4 [9], and YOLOv4-tiny versions. Finally, we provide evaluation results on both static images and video sequences, and some remarks on the discriminative ability of each classifier are presented.

## 2. Related Work

In the field of computer vision, there is often confusion between the concepts of image classification, object localization, and object detection. Applications benefiting from image classification, object localization, and object detection cover a wide range such as social media, security control systems, autonomous driving, and even the most up-to-date SARS-CoV-2 virus spread assessment systems. Image categorization refers to the assignment of an image tag, which identifies the class of the object. On the other hand, spatial detection involves the design of a bounding box around each object of interest in the photograph. Finally, the term object detection includes both of the above concepts, assigning a class tag to each bounding box that is created.

**Object detection and localization models:** The YOLO network [6] is the first CNN model that solves the problem of simultaneous identification and detection of objects in images, with a forward pass. Unlike other models, YOLO treats this problem as a setback rather than a categorization problem. One peculiarity of this network is that it aims mainly at the speed of object recognition. This results in a reduction in recognition accuracy, which is lower than other models, such as Fast-RCNN [10] or Faster-RCNN [11]. In addition, YOLO uses the entire image during the training and testing process and does not use area-based techniques, such as the sliding window.

Regions-based convolutional neural network (R-CNN) [12] is one of the first to use CNNs to detect objects. R-CNN managed to achieve quite high performance when it made its appearance; however, it had several drawbacks that were improved in later implementations. The main disadvantages of R-CNN include: (i) the training takes place in several stages, (ii) the features extracted from the area suggestions are stored on disk, taking up a large volume and is a very time-consuming process, and (iii) object detection is slow, even when running on the GPU.

Spatial pyramid pooling (SSP-net) [13] is a network structure that can create fixed-length representations regardless of the size and scale of the image. SPP-net is resistant to object distortion and improves all CNN-based classification methods. With this structure, one can calculate feature maps from the image only once and then group the features into arbitrary areas (subimages) to create fixed-length representations for image detection training.

Fast R-CNN [10] is also an object detection model and is an evolution of R-CNN, solving several of its problems. The main change offered by the fast R-CNN model is that instead of feeding the area suggestions to the network, we feed the entire CNN image to create a single map of convolutional features. Fast R-CNN offers better performance in terms of speed, accuracy, and training times compared to previous implementations.

Faster R-CNN [11] is a development of the R-CNN family of models. The algorithm solved many problems of the previous versions by using a new detection network called the Region Proposal Network(RPN). Faster R-CNN achieved higher efficiency and much shorter object detection times than the aforementioned models, making it more efficient and able to detect objects even in realtime.

Mask R-CNN [14] is a very effective, accurate, and flexible solution to the problem of object detection. The specific architecture creates frames with which it surrounds the detected objects and at the same time creates segmentation masks for these objects, thus providing their exact outline. Finally, Mask R-CNN provides convenience in the field of education.

Single shot multibox detector (SSD) [15] is a method for detecting objects that uses only one neural network. Instead of first creating area suggestions and then categorizing them, as is performed in the R-CNN algorithm, the SSD simultaneously performs these functions on a single network. This is also a feature of the method that makes training quite easy.

**Face-mask detection models:** There has been a plethora of recent methods [16,17,18,19,20] that explore neural networks to perform deep face recognition tasks under the existence of facial masks.

The masked face recognition (MFR) challenge [17] explored the performance of different face recognition models under the existence of facial masks. The MFR challenge contains two main tracks, namely, the InsightFace track and the WebFace260M track [16]. Each of the two tracks is a collection of large-scale face data sets that includes images of masked and unmasked adults and children with multiracial captures.

If deployed correctly, the face-mask detector may be used to help ensure the safety of the public. To this end, Zhang et al. [21] implemented a face-mask detection model including 500 faces with masks and 500 faces without masks. Moreover, Yang et al. [22] deployed YOLOv5 [23] as an object detector model to train a supervised model that recognizes persons wearing masks in public places. Du et al. [24] defined the problem of masked face recognition in the near-infrared to visible space and built a semi-siamese network to cope with the information from the two domains. The authors stated that the masked face recognition in the near-infrared probe images is a quite difficult challenge.

Mask occlusion may lead to obstruction of the feature structure of the face as certain parts of the face are hidden; thus, detecting facial masks is an important step for effectively recognizing masked and occluded faces in the wild. Wang and Kim [25] trained a convolutional neural network in real and simulated data of masked and unmasked faces to alleviate the problem of facial-mask detection. A novel approach that addressed the problem of masked face recognition by extracting deep features from the unmasked regions of the face and then using the bag-of-features paradigm to the learned feature maps was proposed in [26]. Finally, the visual attention mechanism was also employed in [27] to enhance the recognition accuracy by focusing on the regions around the eyes.

Generative adversarial networks (GANs) have also been used to train robust models for the identity-preserved masked face recognition task [19,28,29,30]. Geng et al. [28] deployed a GAN-based method to generate masked faces and trained a domain constrained loss to bring the inpainted masked faces as close as possible to their corresponding identity full faces. In the same spirit, the work of Ge et al. [29] proposed an identity-preserved inpainting model based on GANs to alleviate the task of occluded face recognition. To cope with the lack of the existence of a large-scale training and test data with ground truth for the tasks of mask-face detection and recognition, Ding et al. [30] created two datasets of synthetic masked face images designed for mask-face detection and recognition, which contain 400 pairs of 200 identities for verification, and 4916 images of 669 identities for identification.

**Difference to previous face-mask detection datasets:** This paper presents the methodology for constructing a new image dataset consisting of people with or without a mask in indoor and outdoor environments. Existing datasets of masked people identification (e.g., masked face recognition (MFR) challenge [17], Zhang et al. [21], and Ding et al. [30]) created two datasets of synthetic masked face images designed for mask-face detection and consist of a significantly fewer number of individual subjects and images (i.e., 500 faces with masks and 500 faces without masks) mostly captured under controlled pose and illumination circumstances. In comparison to these datasets, the proposed FaceMask dataset contains thousands of faces with various face poses and illuminations and people in indoor and outdoor places, individual faces, partially occluded faces, and crowded images with blurred faces that play a vital role in the success of masked face recognition algorithms. Moreover, the collected images depict people of different ages and nationalities who may or may not wear a face mask. In addition, there was a need to cover a wide range of mask detection cases in the wild. For this reason, all images were selected to show blurry and distant faces of people in either indoor or outdoor environments, while at the same time the faces can be individual or there can be an overlap of other objects or persons. The diversity of the data tries to approach the real-world conditions that the detector will be called to cope with.

## 3. Image Database

A reliable annotated image dataset is substantial for the implementation of an efficient object detector that is based on deep learning techniques. The basic steps that we have followed for the construction of our image dataset are described in the following paragraphs.

### 3.1. Data Collection

The images of our database were collected through extensive search in Google images, using keywords such as “people wearing face mask”, “crowds during coronavirus”, and “coronavirus transportation”. The results of our search are 4866 images containing people of several ages wearing or not wearing a mask on their faces. The selected images depict people in indoor and outdoor places, individual faces, partially occluded faces, and crowded images with blurred faces. The number of images and their contents are presented in Table 1.

**Figure 1:**
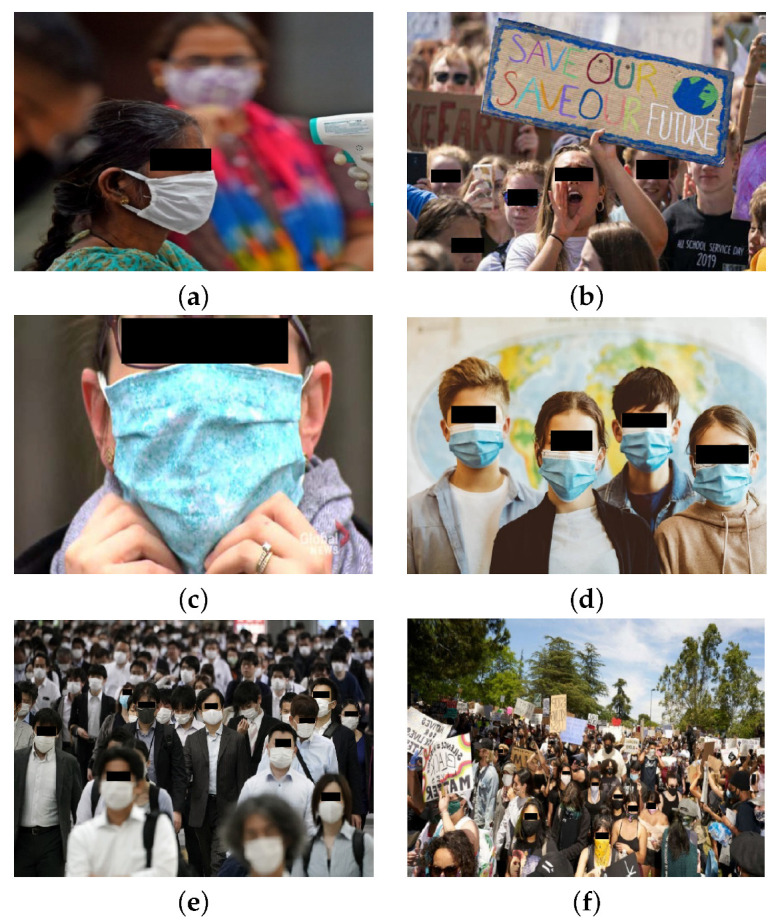
Indicative images of the FaceMask database. (**a**) Blurred faces, (**b**) obscured faces, (**c**) cropped faces, (**d**) individual faces, and (**e**,**f**) crowded images containing blurred, distant, and overlapped faces.

Thus, the individual images contain faces that are not overlapped or occluded. The partially occluded images contain faces that are overlapped by other faces or objects and faces lying at the border of the image, which are not depicted completely and only a part of the face is included. In crowded images are classified those images that contain more than 10 persons. Finally, in the blurred images, the faces are obscured due to low image resolution, lens misfocus, and extensive distance from the camera. It must be noted that an image can belong to more than one of the aforementioned categories; for instance, a crowded image can also contain blurred faces of partially occluded faces. Figure 1 illustrates some representative images included in the FaceMask database.

The images and their corresponding URL were downloaded using the SERP API [31]. The SERP API is an application programming interface (API) that provides access to Google search results and other search engines. Using this API and the resulting JSON files exported per hundred images, we were able to download the image files as well as their URL. To do this, a simple code file was implemented in Javascript using Node.js. A pseudocode of this Javascript Implementation is shown in Algorithm 1. The original Javascript code can be found in the datasets webpage in [5].

 **Algorithm 1:** Algorithm for downloading images and their URLs from the Web. 
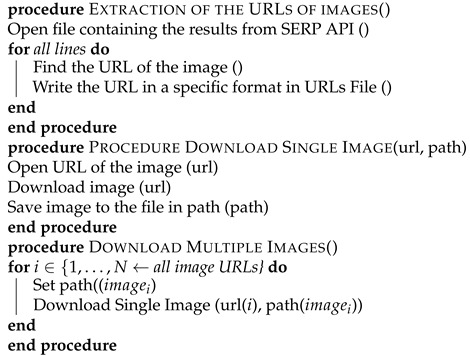


### 3.2. Duplicate Image Removal

Since the selection of images was obtained in an automated manner, in the downloaded dataset, there were several duplicate images with the same content but usually with different sizes. The images that were damaged or did not depict any human faces were deleted and the screening process was followed to ensure that there were no duplicate images.

For this reason, the AntiDupl.NET [32] was used, which is open-source software for the detection and removal of duplicate images. When this process was complete, the associated URLs were also edited, so that the addresses that no longer corresponded to an image are also deleted.

### 3.3. Annotation of the Dataset

For the image annotation process, the LabelImg tool [33] was used. This tool allows saving annotations in XML files using PASCAL VOC format supported by ImageNet [34]. It also supports YOLO format files which are stored in TXT files.

**Figure 2:**
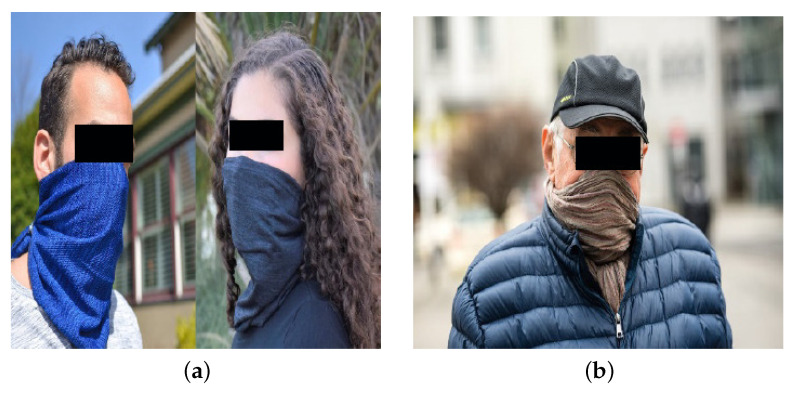
Images in *Mask* category, containing faces that wear (**a**) neckband and (**b**) scarf instead of a mask.

The FaceMask database contains images from two categories, namely, *Mask* and *No_Mask*. In the *Mask* category the images of people wearing a mask are assigned. The mask may cover their nose or not. We have also included all the images depicting people wearing a cloth on the face covering their mouth and nose, such as scarves, or fichu. Furthermore, in the *No_Mask* category, images depicting people wearing a mask in a wrong way, with the mouth uncovered were also included. Figure 2 depicts some examples of the of *Mask* category images.

Apart from the LabelImg tool [33], the annotation process was also supervised by an additional observer who watched the images independently and recorded their labels separately. Disagreement between the LabelImg tool and the observer was resolved by a second observer. It is worth mentioning that the initial annotator and the LabelImg tool disagreed in only 2% of the images of the dataset. Such images may contain faces in which the mouth and/or the nose are not fully covered by the mask or faces that are occluded by scarfs, fichu, neckbands, or other objects. The observers were asked to categorize those images into the corresponding category. Both annotators agreed that when the mouth and/or the nose were not fully covered by the mask the corresponding image should be marked as *No_Mask* category.

**Figure 3:**
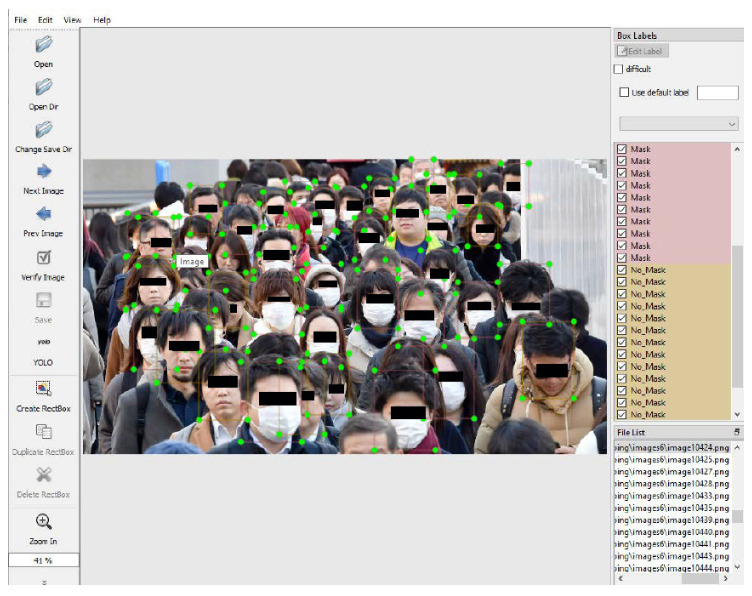
Label assignment using LabelImg in an image with many faces of both categories (*Mask* and *No_Mask*).

Figure 3 depicts an example of label assignment of categories *Mask* and *No_Mask* to an image containing multiple crowed persons in the wild.

## 4. Evaluation of Detection Algorithms Using FaceMasK

We tested several classification schemes on the FaceMask dataset to evaluate their performance on the discrimination of the two classes of images. More specifically we performed experiments with three versions of the YOLO network [6], which is the first model of convolutional neural network that simultaneously addresses the problem of object detection and object localization, with a single forward pass. It is able to recognize objects with high performance and in a fast way, as it takes as input the whole image, avoiding cropping of the image and the use of a sliding window. The YOLO detector is fast and effective, which makes its use attractive in realtime applications.

All experiments were conducted on a graphic workstation with Intel i7-9750H 2.6 GHz CPU (6 cores, 12 threads), 16 GB DDR4 RAM 2666MHz, and Nvidia GeForce RTX 2060 (1920 CUDA cores and 6 GB GDDR6) GPU. In the following paragraphs, a detailed description of each classification scheme is provided.

### 4.1. YOLO

**Figure 4:**
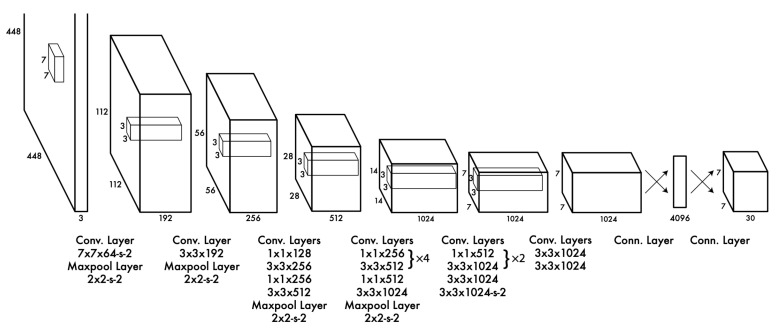
The architecture of YOLO [6].

The architecture of YOLO consists of 24 convolutional layers and 2 fully connected layers (Figure 4). The procedure that is followed includes the separation of the image in a grid of a specific size. The network predicts in every cell of the grid several bounding boxes with the corresponding confidence score, which represents the accuracy that a detected object belongs to the specific bounding box. The confidence score is given by:

(1) $$conf=P\left(object|box=i\right)*IoU_{pred}^{truth}$$

The term $IoU_{pred}^{truth}$ corresponds to the intersection over union, which is a number between zero and one, and defines, for each bounding box, the percentage of overlap between the predicted frames and the ground truth.

In every grid cell, a prediction of the probability Pr(class|object) of the class of the object is provided. In the case that no object is detected in the specific cell, the confidence score is zero, otherwise the confidence score for a given class *i* is given by:

(2) $$\begin{array}{cc} conf_{i} & =P\left(class_{i}|object\right)*P\left(object|box=i\right)*IoU_{pred}^{truth}\\ & =P\left(class_{i}\right)*IoU_{pred}^{truth} \end{array}$$

Finally, in each bounding box, four more predictions are also provided, namely the center (x,y) of the bounding box, its width *w*, and height *h*.

### 4.2. YOLOv3

The first version of YOLO was quickly evolved for the enhancement of its performance. Thus, the third version of YOLO was proposed in [8]. The basic functions of YOLOv3 were the same as YOLO, but it exhibits several different specifications. The first involves the definition of the bounding box. The network predicts four coordinates $t_{x}$, $t_{y}$, $t_{w}$, and $t_{h}$. If a cell offset $\left(c_{x},c_{y}\right)$ occurs, in terms of the upper left corner of the image, and the bounding box prior has width and height $\left(p_{w},p_{h}\right)$, then the predictions are given by [8]:

(3) $$\begin{array}{cc} \; & b_{x}=\sigma \left(t_{x}\right)+c_{x}\\ \; & b_{y}=\sigma \left(t_{y}\right)+c_{y}\\ \; & b_{w}=p_{w}e^{t_{w}}\\ \; & b_{h}=p_{h}e^{t_{h}} \end{array}$$

The objectness score (according to the confidence score) represents an indication of the overlapping of the bounding box and the object. Only one bounding box is assigned to each object, based on the maximum percentage of overlapping of the object (IoU). The loss function of YOLOv3 consists of three parts:

- *Classification loss*: in the case of the detection of an object, for every cell of the grid, the sum of squares of the probabilities that the object belongs to a class is calculated;
- *Localization loss*: the error between the predicted bounding boxes and the ground truth is calculated;
- *Confidence loss*: it measures the objectness of a bounding box, in the cases where an object is either detected or not in this bounding box.

**Figure 5:**
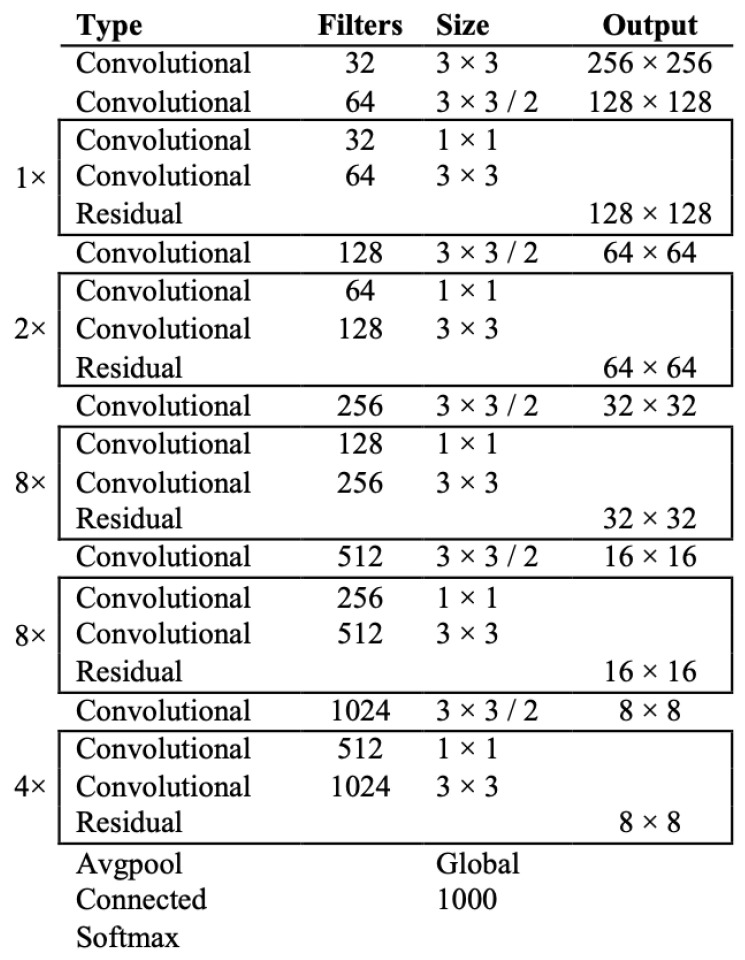
The architecture of Darknet-53 [8].

Another improvement of YOLOv3 is that it provides multilabel classifications, and it uses shortcut connections, for the detection of small objects. Furthermore, a new network for feature extraction is included. This network contains 53 convolutional layers and it is called Darknet-53 [7]. Its architecture is depicted in Figure 5.

As described in [8], the limitations of YOLOv3 are (a) anchor box x, y offset predictions, (b) linear x, y predictions instead of logistic, (c) focal loss, and (d) dual IOU thresholds and truth assignment.

### 4.3. YOLOv4 and YOLOv4-Tiny

YOLOv4 [9] is an improved version of YOLOv3 in terms of two metrics, which are extensively used for the evaluation of the performance of a classification algorithm: the Average Precision and the processing time (frames per second). In YOLOv4, the Cross-Stage-Partial Darknet-53(CSPDarknet53) is used as a feature extractor, and its training can be easily performed in a single GPU. Furthermore, the techniques Bag-Of-Freebies (BoF) and Bag-Of-Specials (BoS) were developed in the detector and the backbone part of the network. These techniques aim to increase of the accuracy of the predictions. Table 2 shows the parameter set used for training YOLOv4.

In addition, YOLOv4-tiny is based on a light computational version of YOLOv4, which results in faster detection of the objects. This is achieved because the architecture of YOLOv4-tiny is simpler. Thus, the convolutional layers in the backbone part of the network are compressed. Furthermore, there are only two (instead of three) YOLO layers, and it uses fewer anchor boxes for the prediction of the bounding boxes. However, the reduction in computational time usually introduces limitations in the performance of the network, but it still presents comparable results with the other versions of YOLO.

## 4.4. Training

We trained the YOLO detector through Darknet, following the documentation instructions for the installation. In each configuration of YOLO, we used the parameters of Table 2. We use pretrained weights for the convolutional layers.

In YOLOv4 and YOLOv4-tiny the IoU loss function was also used, which represents the overlapping of the initial bounding box that contains the object and the predicted bounding box.

For the evaluation of the performance, the *Precision* and *Recall* metrics were used, given by:

(4) $$Precision=\frac{TP}{TP+FP}$$

(5) $$Recall=\frac{TP}{TP+FN}$$

where TP, FP, and FN are the true positive, the false positive, and the false negative predictions for each class. The Precision-Recall Curve (PR-curve) of eleven points was then constructed and the area under the curve was calculated, which corresponds to the mean average precision (mAP). Furthermore, in each bounding box, the intersection over union (IoU) between the ground truth bounding box and the predicted bounding box was calculated. IoU defines the percentage of overlap between the delimitation frames provided and the initials. The mAP was estimated with IoU threshold=0.5, following the PASCAL VOC challenge [35].

## 5. Experiments and Results

We performed several experiments for the training of YOLOv3, YOLOv4, and YOLOv4-tiny. Furthermore, a k-fold cross-validation scheme was used for the evaluation of the performance of the method.

More specifically, the image set is composed of 4866 images, which were randomly separated into three subsets, the training set, the validation set, and the test set. In Table 3, the number of faces in each subset and their class is provided. In each experiment the metrics mAP, Average IoU (for threshold=50%), and AP (Average Precision) for the classes *Mask* and *No_Mask* are calculated, corresponding to different value weights, which were calculated in 1000 iterations and present the highest performance.

**Figure 6:**
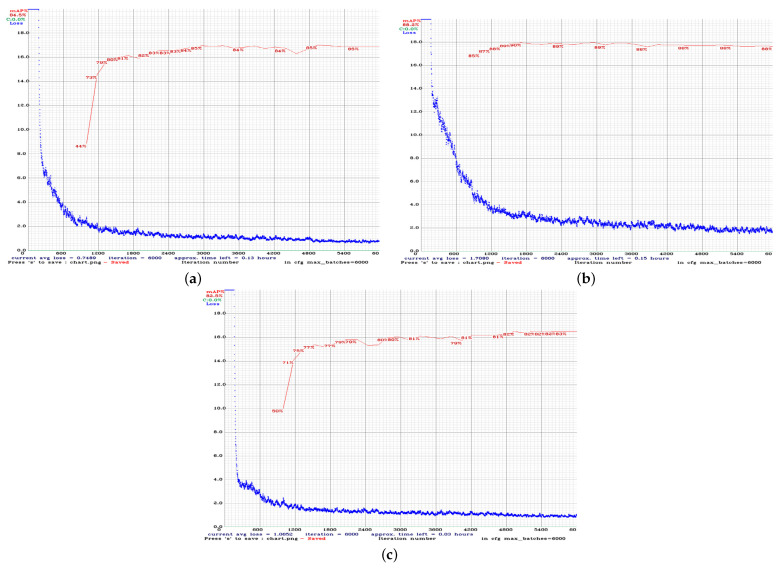
Performances of (**a**) YOLOv3, (**b**) YOLOv4, and (**c**) YOLOv4-tiny models in terms of average loss and mAP.

The training procedure for all models was performed for 6000 iterations. The average loss and mAP during the iterations for YOLOv3, YOLOv4, and YOLOv4-tiny models are depicted in Figure 6, where the mAP metric was calculated on the validation set every four epochs. Each epoch was determined as a fraction of (images in the training dataset)/batch_size. This metric is considered the most important metric, based on the documentation of the Darknet. After 1000 iterations, we saved the weight values and the values with the highest performance (Table 4). The same procedure was followed for YOLOv4 (Table 5) and YOLOv4-tiny (Table 6). For YOLOv3 and YOLOv4 the learning rate was set to 0.001, and for YOLOv4-tiny the learning rate was set to 0.00261.

### 5.1. Comparison between Different Models

**Figure 7:**
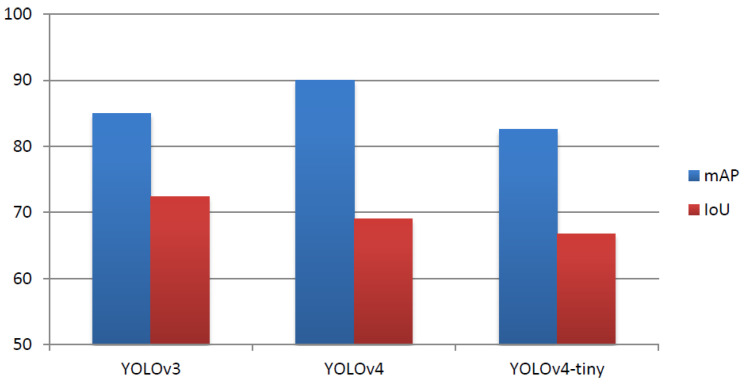
IoU and mAP indices for the three models.

As verified by the experimental results, we observe that considering Average Precision, the performance of YOLOv4 was higher by 5.09% and 7.48% than the corresponding performance of YOLOv3 and YOLOv4-tiny, respectively. Furthermore, YOLOv3 performed better by 2.39% in terms of mAP than YOLOv4-tiny, and it exhibited higher performance than all models in terms of Average IoU. More specifically, its performance was 3.39% higher than YOLOv4 and by 5.64% higher than YOLOv4-tiny in terms of IoU. Finally, the performance in terms of average loss after 6000 iterations for YOLOv3, YOLOv4, and YOLOv4-tiny was 0.7489, 1.7080, and 1.0652, respectively. Figure 7 depicts the comparison of the three models in terms of IoU and mAP metrics.

**Figure 8:**
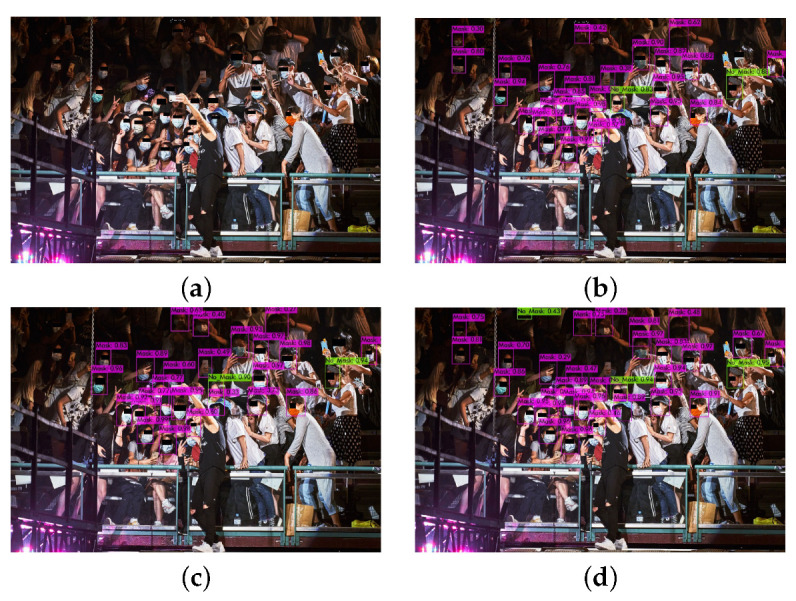
(**a**) Initial image and the classification results of (**b**) YOLOv3, (**c**) YOLOv4, and (**d**) YOLOv4-tiny.

**Figure 9:**
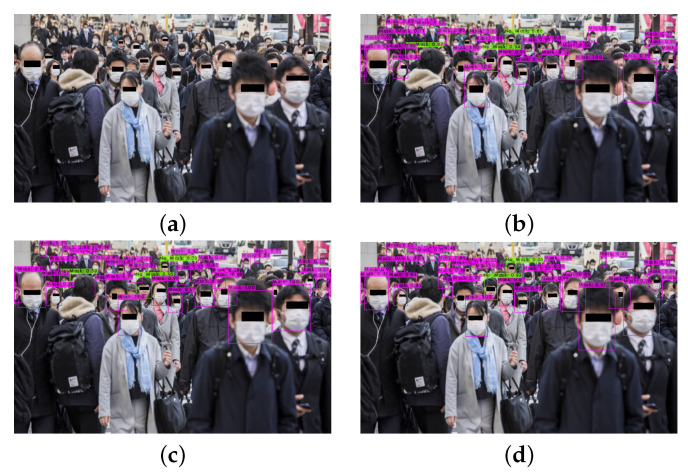
(**a**) Initial image and the classification results of (**b**) YOLOv3, (**c**) YOLOv4, and (**d**) YOLOv4-tiny.

If we take for granted that the metric that characterizes the performance of each network is the mAP metric, we can conclude that YOLOv4 outperforms all the other versions of YOLO. This can be explained because it includes the BoF and BoS techniques in the backbone and in the neck part of the network, and it uses the CSPDarknet53 method for the extraction of the features. These techniques are not included in the previous version of YOLOv3. Furthermore, YOLOv4-tiny converges faster than YOLOv4, and this leads to a decrease in precision. Some examples of the classification results of the different models in real images are depicted in Figure 8 and Figure 9.

In addition, for the evaluation of the data set, a two-fold cross-validation scheme was performed, and the comparison of the results is presented in Table 7. As we can see, the models exhibited higher performance with the two-fold cross-validation scheme, in terms of average mAP. Observing the results, we can conclude that with this random data partition, we achieve better performance at an average accuracy of 1.66% in YOLOv3, 0.65% in YOLOv4, and 0.65% in YOLOv4-tiny models. Since as mentioned above, the mAP value is the one that determines to a greater extent the performance, we conclude that with the two-fold cross-validation partitioning process the results produced were better than with the original set data.

The evaluation of our data shows that the classification task is quite accurate in the whole range of the dataset, and regardless of the division, the obtained results are equally accurate and satisfying. The same conclusion is reached for the network, which achieves good performance even in data that are unknown to it.

### 5.2. Failure-Cases Analysis

**Figure 10:**
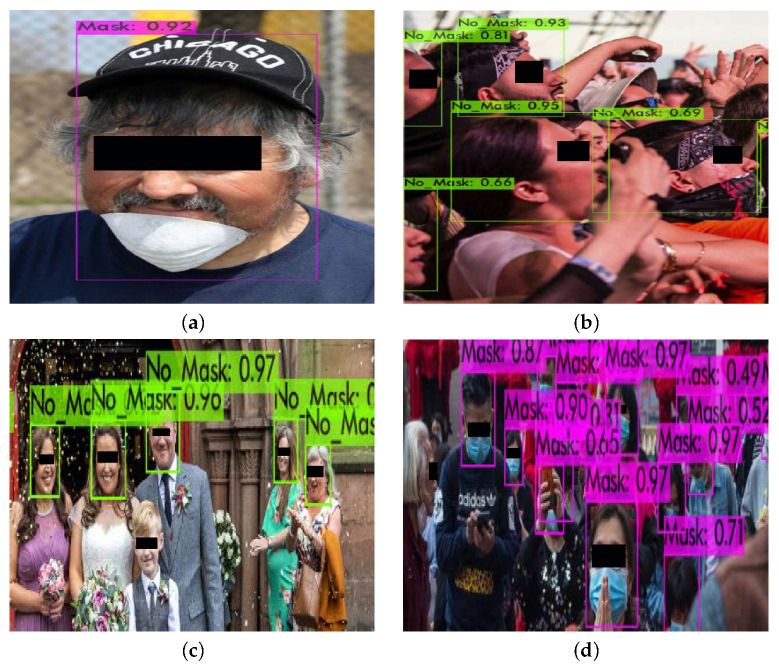
Classification failure cases. These images may contain (**a**) faces in which the mouth and the nose are uncovered, faces with (**b**) a neckband, (**c**) faces that are not detected, and (**d**) side faces at the border of the image.

It must be noted that the cases of misclassification are commonly observed in images that contain people that are not wearing the mask correctly or the mask is different than the specific type of mask (i.e., surgical mask). Furthermore, images containing faces that are highly occluded and images of low resolution may not be correctly classified to the underlying category. Figure 10 demonstrates some failure case examples with regard to the suggested categories.

To cope with these failure cases, the evaluation policy should be revised to take into account examples that belong to classes that may hold similar semantic characteristics, from the database perspective. Furthermore, from the training model perspective, it is important to improve the model itself so as to take into account the inter-class similarity. When there are examples that obtain high-prediction weights, then similar examples that may contain faces in which the mouth and/or the nose are not fully covered by the mask, or faces that are occluded by scarfs, neckbands, or other objects may also be assigned with high-prediction weights.

Moreover, there are cases where it is hard to say that the prediction model is incorrect because faces may not be salient in the image. These cases are considered to be supplemented, thus a revision of the original dataset or the evaluation policy is necessary.

There are also challenging cases that are difficult to predict the underlying category even by humans. For example, faces may be covered by a neckband used as a mask as shown in Figure 10b; thus, it is quite difficult to identify the correct class without contextual knowledge.

Finally, incorrect ground truth images in the dataset may also lead to mis-classification cases. These incorrect ground truth images should be complemented by the strict revision of the dataset. Images that are out of the regular range of imaging distribution (i.e., images with high distortion of illumination and motion blur) may also lead to incorrect classification. To classify these images correctly, the FaceMask dataset should be extended and training models should be improved so that during training images with irregular illumination and motion blur are also present.

## 6. Ethical Concerns

In recent years, several concerns about the misuse of facial recognition algorithms have emerged. There is both confusion and exaggeration over potential risks, while questions over ethical concerns about invasion of this technology in daily life and privacy are also valid. In this research, we used images available online as well as their URLs to train well-known face-mask recognition algorithms. The dataset is made publicly available consisting only of the corresponding image URLs and NOT the original images. The data are preprocessed compressed into a binary record, and only the image URLs are publicly available. Our private image data will not be released to the public to avoid the data privacy problem.

## 7. Conclusions

In this work, the publicly available FaceMask image dataset was introduced, which was created for the recent requirement of the automated detection of people wearing a mask in crowded places, due to the COVID-19 pandemic. It contains 4866 images of two categories, *Mask* and *No_Mask*, which were carefully selected in order to correspond to real conditions. We have used three versions of the YOLO network, i.e., the YOLOv3, YOLOv4, and YOLOv4-tiny for the automated detection of people wearing masks using the FaceMask image dataset, and the results indicate that YOLOv4 presents the best performance. The results of the classification schemes provide a reference point for the evaluation of future approaches for the automated identification of people wearing a mask.

As future work, we intend to extend our image dataset with images containing people of different nationalities, in order to enhance the performance of the correct identification of each face. Furthermore, we can include images of different kinds of masks except for the surgical mask, such as helms or shield masks. In addition, we intend to comprise another class of images, containing the images which depict people wearing a mask in a correct way. Finally, the installation of the trained models in smartphones and the use of input images from the camera preview will lead to realtime classification.

## Figures and Tables

**Table 1:** FaceMask Contents.

Content	No. Images	Description
Individual	3529	Single faces with no overlap or occlusions
Occluded	1307	Partially occluded faces
Crowded	994	More than 10 faces
Blurred	1068	Obscure faces

**Table 2:** Parameter Setting.

**Batch**	64	**Steps**	4800, 5400
**Subdivisions**	16	**Width × Height**	416 × 416
**MaxBatches**	6000	**YOLO Filters**	21

**Table 3:** Contents of training, validation, and test set.

Class	Training Set	Validation Set	Test Set
*Mask*	9328	3819	2272
*No_Mask*	8169	1585	2508
**Total**	17,497	5404	4780

**Table 4:** Performance of YOLOv3.

Iterations	mAP	Average IoU	AP *Mask*	AP *No_Mask*
1000	44.35	33.45	33.77	54.93
2000	81.07	66.09	79.93	82.21
3000	82.72	68.12	81.86	83.57
4000	84.21	68.38	83.19	85.24
5000	84.75	72.18	83.80	85.71
6000	84.56	72.89	83.56	85.55
**Best**	84.95	72.38	83.85	86.05

**Table 5:** Performance of YOLOv4.

Iterations	mAP	Average IoU	AP *Mask*	AP *No_Mask*
1000	85.17	60.83	85.78	84.57
2000	90.04	68.99	89.98	90.10
3000	89.68	71.13	89.14	90.22
4000	89.79	69.89	89.12	90.46
5000	88.63	74.50	88.13	89.13
6000	88.21	75.06	87.94	88.48
**Best**	90.04	68.99	89.98	90.10

**Table 6:** Performance of YOLOv4-tiny.

Iterations	mAP	Average IoU	AP *Mask*	AP *No_Mask*
1000	50.05	24.56	41.82	58.27
2000	78.05	62.24	77.54	78.56
3000	80.04	61.51	79.47	80.61
4000	80.88	62.98	80.19	81.56
5000	82.51	66.91	81.62	83.39
6000	82.56	67.24	81.55	83.57
**Best**	82.56	66.74	81.58	83.55

**Table 7:** Comparison of initial training and two-fold cross validation scheme.

YOLO Versions	mAP of Initial Training	mAP of 2-Fold Cross Validation (Mean ± Std)
YOLOv3	84.95	86.65±1.66
YOLOv4	90.04	90.69±0.65
YOLOv4-tiny	82.56	83.21±1.65

## Data Availability

The reported FaceMask dataset is publicly available at https://mvrigkas.github.io/FaceMaskDataset/ (accessed on 22 October 2021).
